# Particle Reirradiation of Malignant Epithelial and Neuroectodermal Sinonasal Tumors: A Case Series from CNAO

**DOI:** 10.3390/jcm12072624

**Published:** 2023-03-31

**Authors:** Barbara Vischioni, Rossana Ingargiola, Maria Bonora, Sara Ronchi, Anna Maria Camarda, Stefania Russo, Eleonora Rossi, Giuseppe Magro, Alfredo Mirandola, Ester Orlandi

**Affiliations:** 1 Radiation Oncology Unit, Clinical Department, National Center for Oncological Hadrontherapy (CNAO), 27100 Pavia, Italy; 2 Medical Physics Unit, Clinical Department, National Center for Oncological Hadrontherapy (CNAO), 27100 Pavia, Italy

**Keywords:** sinonasal cancer, reirradiation, particle therapy, carbon ion, proton therapy, head and neck

## Abstract

Sinonasal cancers (SNCs) are rare and heterogeneous in histology and biological behavior. The prognosis is generally unfavorable, especially in inoperable cases. In recent years, for some histologies, such as undifferentiated sinonasal carcinoma (SNUC), multimodal treatment with a combination of induction chemotherapy, surgery, and chemo/radiotherapy (RT) has improved the prognosis. Nevertheless, still about half of the patients treated incur a recurrence, in most of the cases at the local site. Surgery with and without RT is usually the treatment choice in cases of recurrence after previous RT in combination with systemic therapy or RT in a histology-driven fashion. In the case of inoperable disease or contraindications to surgery, RT is still a valid treatment option. In this context, hadron therapy with protons (PT) or carbon ions (CIRT) is often preferred due to the physical and biological characteristics of charged particles, allowing the administration of high doses to the tumor target while sparing the surrounding healthy tissues and potentially limiting the side effects due to the high cumulative dose. In the absence of a standard of care for the recurrent setting, we aimed to investigate the role of re-RT with PT or CIRT. We retrospectively analysed 15 patients with recurrent, previously irradiated, SNCs treated at our institution between 2013 and 2020. Local control (LC) and overall survival (OS) were estimated by the Kaplan–Meier method. Acute and late toxicities were scored according to the National Cancer Institute’s Common Terminology Criteria for Adverse Events CTCAE version 5.0. A total of 13 patients received CIRT and 2 patients received PT. The median re-RT dose was 54 GyRBE (range 45–64 GyRBE) delivered in 3 or 4 GyRBE/fr (fraction) for the CIRT, and 2 Gy RBE/fr for the PT schedule. LC was 44% at the 1-year follow-up and 35.2% at the 3-year follow-up. OS at 1 and 3 years were 92.9% and 38.2%, respectively. Fourteen patients developed G1–G2 acute toxicity (dermatitis and mucositis), and no patients developed G3–G5. Regarding late toxicity, 10 patients encountered at maximum G1–2 events, and 4 did not experience any toxicity. Only for one patient G3 late toxicity was reported (dysphagia requiring a percutaneous endoscopic gastrostomy).

## 1. Introduction 

Sinonasal cancers (SNCs) are very rare and heterogeneous diseases [1]. Although the primary management depends on the histological type, the global performance status, the pathway of local and regional spread, the foreseen sequelae of the treatment, and the availability of a multidisciplinary and experienced team, the most common therapeutic strategy relies on the combination of surgery, radiotherapy (RT), and chemotherapy [2,3]. In the last few decades, the advances in surgery and RT techniques, such as particle therapy (including both RT with protons PT and carbon ions CIRT) and the integration of histologically driven systemic treatment, have allowed for the achievement of better survival outcomes for locally advanced SNCs [4]. However, SNCs locally recur in 32–80% of treated cases, varying with a substantial range according to histology, stage at primary diagnosis, and other factors [5]. Most recurrences manifest within 2–5 years after primary treatment [6], but some specific tumor types, such olfactory neuroblastoma (ONB), may recur even after several years [7].

The management of locoregional recurrences still represents a challenge because of the anatomic complexity of the sinonasal region, the variable biological behavior of the different histologies, and the tissue alterations induced by previous treatments. Consequently, it is of paramount importance that any form of re-treatment must be managed by an experienced multidisciplinary head and neck oncologic team with all the necessary manpower and equipment available. In general, like in the primary setting, surgery is the mainstay of treatment [8]. However, only few patients are likely to be suitable for salvage surgery with radicality after restaging. In this scenario, non-surgical options, such as re-irradiation (re-RT) with photons or particles RT, may play a role, although the potential toxicity and benefits need to be considered on a case-by-case basis. PT and CIRT are frequently considered for re-RT due to their favorable physical characteristics that allow for the delivery of high doses to the tumor target, sparing the surrounding normal tissues [9]. In addition, CIRT, in consideration of the much higher radiobiological effectiveness in comparison to conventional photon RT, reduced oxygen enhancement ratio (OER) and the induction of clustered DNA damages, is potentially capable of effectively killing radioresistant tumor clones [10,11], such as the ones deriving from several hystotypes from the sinonasal region. Based on these premises, particle RT could be a valuable option, not only in the primary setting as shown in a recent metanalysis on 2282 SNCs from the real world [12], but even for previously irradiated recurrences. In a recent retrospective cohort study with 242 recurrent primary head and neck squamous cell carcinomas retreated with PT with a 2-year overall survival (OS) of 53%, it has been suggested that patients retreated with aggressive PT (median re-RT dose of 70 cobalt gray equivalent CGE for the fractionated cohort) have a survival advantage compared to photon-based re-RT, although with a high risk of early and late treatment-related complications [13]. Furthermore, Gordon et al. reported the feasibility of re-RT even in a cohort of unresectable recurrent SNCs with 1/2 years of local control (LC), progression-free survival (PFS), and an OS of 52.6/21.0, 21.9/10.9, and 73.4/8.4% [14], and 16.6% had late severe side effects and there was one re-RT related death.

Little data have been published on re-RT with PT/CIRT for recurrent SNCs [15,16,17], in particular for epithelial nonglandular and neuroectodermal tumors, and, to date, we are still far from defining the best re-RT approach, both in terms of RT techniques and fractionation. In this retrospective study, we aim to investigate the clinical outcome and toxicity of PT/CIRT re-RT in a mono-institutional cohort of patients with recurrent SNCs previously treated with photon RT.

## 2. Materials and Methods

### 2.1. Study Population

We present a retrospective study on 15 patients with recurrent previously irradiated SNCs treated with particle therapy (PT or CIRT) at CNAO from 2013 up to 2020. The present study was approved by the CNAO Ethical Committee. Inclusion criteria were as follows: (a) diagnosis of recurrent malignant epithelial SNCs after one previous RT course including ONB (salivary gland type carcinoma; sarcoma and mucosal melanoma were excluded) without metastatic disease at the time of re-RT; (b) histologically confirmed recurrence after physical examination, magnetic resonance imaging (MRI), and/or positron emission tomography with 2-deoxy-2-[fluorine-18]fluoro-d-glucose/CT (18F-FDG PET/CT); (c) previous curative or postoperative RT up to a total biological equivalent dose (EQD2) of at least 50 Gy with intensity modulated RT (IMRT) with conventional fractionation or stereotactic RT; (d) disease-free interval of at least 6 months between the two courses of RT; (e) recurrent tumor with a ≥50% overlap with a previously irradiated area; (f) minimum follow-up after re-RT of 6 months; (g) age > 18 years; and (h) Karnofsky performance status ≥ 60.

Exclusion criteria were as follows: (a) concomitant chemotherapy only if CIRT was indicated; (b) missing data and dose report about previous RT plan; and (c) presence of metal implants not allowing correct dose calculation. All patients were staged at primary diagnosis adopting the most recent TNM staging classification according to the American Joint Committee of Cancer and International Union Against Cancer (AJCC/UICC) guidelines (8th Edition).

### 2.2. Re-RT Work out and Patient’s Follow-Up

All patients performed a complete restaging with baseline MRI and total body contrast-enhanced CT or FDG PET/CT before PT or CIRT. Treatment procedure for each head and neck patient at CNAO includes a CT scan simulation (2 mm slice-thickness) with personalized positioning systems with a thermoplastic mask. Three-Tesla MRI images with contrast medium were acquired in the same set-up conditions as the simulation CT [18]. In case of contraindication to MRI, a CT scan with contrast medium was performed. Target volumes and organs at risk (OARs) were contoured based on CT scan and MRI fusion images. Target volumes included the gross tumor volume (GTV) with a geometric margin of 3–5 mm (adapted on anatomical barriers and OARs) to obtain a clinical target volume (CTV). For each patient, digital imaging and communication in medicine (DICOM) files or data extracted from printed CT images of previous RT were collected and analyzed. Cumulative dose volume histograms (DVH) and dose distributions were recalculated based on deformable registrations between CT acquisitions referring, respectively, to the first photon RT treatment course and the particle re-RT. An estimate of the cumulative EQD2 from the planned re-RT and the first photon RT course was calculated adopting a α/β ratio of 3 for radioresistant tumors. Constraints to OARs were set considering the cumulative EQD2 by using the QUANTEC reports, except for CIRT re-RT, where constraints for optic nerves, brainstem, and spinal cord were previously described [19,20]. Treatment plans were optimized with Syngo PT Treatment Planning System (TPS) (Siemens AG Healthcare, Erlangen, Germany) until October 2019 and, afterwards, with the Raystation TPS (Raysearch, Stockolm, Sweden).

All patients have been clinically evaluated at baseline, during RT, at treatment completion, and every 3 months for the first 2 years after the end of treatment and then at 6-month intervals with a clinical visit and local imaging with MRI (or CT in case of contraindication to MRI). Acute and late toxicities were scored according to the National Cancer Institute’s Common Terminology Criteria for Adverse Events CTCAE version 5.0.

### 2.3. Statistical Analysis

The follow-up time was calculated from the end of treatment to the last clinical evaluation. LC was defined as the absence of tumor growth of the treated lesion after re-RT. PFS was defined as the absence of locoregional or distant failure. The OS was calculated from the end of re-RT to the time of the last follow-up visit (or available clinical update) or the date of death. Survival curves were estimated by the Kaplan–Meier method and log-rank tests were used for comparison. Outcomes were evaluated as percentage with the appropriate 95% confidence interval (CI). Statistical analyses were performed by the MedCalc Statistical Software version 19.2.6 (MedCalc Software bv, Ostend, Belgium).

## 3. Results

### 3.1. Patients and Treatment Characteristics at the Time of the Primary Tumor Diagnosis

A total of 15 patients (13 males and 2 females) met the inclusion criteria. Patients and tumor characteristics at the primary SNCs diagnosis and treatment are summarized in Table 1. Median age at first diagnosis was 58 years (range 32–84). Primary tumors were mainly located at the nasal fossa in 46.7%, at the ethmoid in six cases (40%) and at the maxillary sinus in two cases (13.3%). A total of 7 out of 15 patients were intestinal-type carcinoma (ITAC), 3 were ONB, 2 squamous cell carcinoma (SCC), 2 sinonasal undifferentiated carcinoma (SNUC), and 1 adenocarcinoma. Treatment at first diagnosis was surgery followed by adjuvant RT in eight cases, exclusive RT in five cases, and surgery alone in two patients (with first RT performed at the first relapse time). A chemotherapy regimen was combined with RT in three patients. First, RT course was delivered with photon IMRT in 14 cases and with CyberKnife in 1 case with a median dose of 60 Gy. All the patients received one RT course before the re-RT at CNAO.

### 3.2. Patients and Treatment Characteristics at the Time of Particle Re-RT

Patients and treatment characteristics at the time of particle re-RT are summarized in Table 2. A total of 7 (46.6%) out of 15 patients underwent re-RT at the second relapse, 5 at the first relapse, and 3 at the third relapse. Median time to first relapse was 20 months (range 3–107). Median age at re-RT was 61.5 years (range 38–87). The median time from first RT course to re-RT was 37 months (range 10–213). Thirteen patients were reirradiated with CIRT and two with PT. Median re-RT dose was 54 GyRBE (range 45–64) delivered in 3 or 4 GyRBE/fr (fraction) for the CIRT and 2 GyRBE/fr for the PT schedule. Median EQD2 was 63GyRBE. In 60% of cases, the relapse occurred in the field of the previous RT plan and 40% at the field borders. Median GTV volume was 36.41 cc (range 3.4–122.89).

### 3.3. Patients’ Outcome after Particle Re-RT

**Figure 1:**
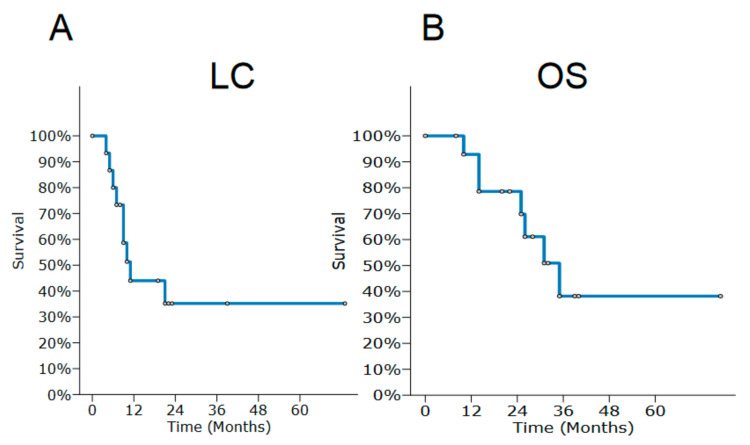
Kaplan–Meyer curve for local control (LC) in the series with patients at risk at 6, 12, 24, and 36 months of 13, 6, 2, and 2, respectively (**A**). Kaplan–Meyer curve for overall survival (OS) with patients at risk at 6, 12, 24, and 36 months of 15, 14, 9, and 3, respectively (**B**). Censored patients are marked with a circle.

After a median follow-up time of 22 months (range 6–95), LC was 44% (95% CI 17.4–70.6) at 1 year and 35.2% at the 2- and 3-year follow-up (95% CI 10.1–66.1) (Figure 1A). Median LC time was 11 months (95% CI 6–21), and median PFS was 10 months (95% CI 5–17). PFS was 33% at 1 year (95% CI 8.9–57.7) and 17.8% (95% CI 0–38.8) at the 2- and 3-year follow-up. Among the 12 patients experiencing disease progression after treatment, 9 developed local failure, 2 had distant metastasis, and 1 had both. Local failure was managed with surgery in two cases, chemotherapy alone in four, one patient was lost to follow-up after relapse, and two patients died without further oncologic treatments. OS at 1, 2, and 3 years were 92.9% (95% CI 79.1–100), 78.6% CI (56.6–100), and 38.2% (95%CI 6.8–69.6), respectively (Figure 1B). Median OS was 35 months (95 CI% 14–35). At the time of the analysis, seven patients were dead from disease, four were alive without disease, three had developed distant metastasis, and one was lost to follow-up. For all the patients except one who was G0, acute toxicity was registered and collected (Table 2 with details in Table 3). For seven patients, maximum acute toxicity reported was G2, seven patients had G1, and no patients developed G3–G4. All the patients except one experienced at least a moderate dermatitis and oral/nasal mucositis requiring local or anti-inflammatory medical treatments; however, they all resolved within 3 months after the end of RT. Regarding late toxicity (Table 2 with details in Table 3), 10 patients developed, at maximum, G1–2 events and 4 did not experience any toxicity. These were mild toxic effects not interfering with daily patient routine, requiring only sometimes local or medical interventions. Only for one patient was there a late G3 dysphagia requiring a percutaneous endoscopic gastrostomy PEG reported at 5 months after the end of re-RT to maintain adequate patient weight. After a rehabilitation course, the patient started to take food orally but had to keep the PEG during the rest of his follow-up.

## 4. Discussion

Our study investigates oncologic outcomes and the toxicity profile of a small series of patients with recurrent SNCs who underwent re-RT with particle therapy at CNAO after a first photon RT course to assess the feasibility of re-RT with hadron therapy. With the limitation of the heterogeneity and the small patient cohort, we present comparable outcome data to previously published reports and provide evidence for the management of recurrent SNCs with particle therapy.

In a re-RT setting, when surgery is not indicated, modern RT techniques are recommended since they allow optimal treatment plans with high-dose conformity (IMRT, stereotactic RT, and particle therapy) with sparing of normal surrounding tissues, and thus increasing the therapeutic ratio. Takiar et al. suggested to consider radical re-RT for relapsed patients when no surgery is possible, when previous RT has been delivered at least 6 months before, and when there is the possibility to deliver at least 60 GyRBE safely based on the analysis of the dose that OARs can tolerate after the first RT course [21]. In addition, Takiar recommended the use of PT in case photon RT is not enough to spare normal organs [21]. Palliative use of particle RT is beyond the scope of particle therapy. When re-RT is offered with palliative intent, photon RT is preferred, also in consideration of the high cost and the paucity of particle treatment centers where research is active to assess appropriateness of indications, and cost-effectiveness analysis are currently ongoing [22].

Preclinical studies have already shown the dosimetric advantages not only for PT in sparing normal tissues [23] but also for CIRT compared to other modern and advanced photon techniques [24]. However, whether the dosimetric advantages can be translated into a clinical benefit for the patients is still an open question since well-designed prospective clinical trials assessing the safety and efficacy of re-RT with particle therapy are lacking and only small retrospective series with heterogeneous treatment sites and modalities are available. To our knowledge, our series, although very small, is the only one available in the literature including exclusively recurrent SNCs reirradiated either with PT or CIRT. Only subanalyses are available on re-RT patients after PT from larger SNCs series including both naïve and previously irradiated patients [15,17]. Our series is small since SNCs are rare tumors and often managed with further surgery after a first primary treatment course that most of the time includes surgery and RT. Same as for other re-RT series after photon RT in the head and neck [25] and SNCs [26,27], our cohort is heterogeneous for histologies, treatment sites, and modalities aiming at including bad prognosis epithelial SNCs (mainly SCC and ITAC) for which research in the primary setting is also ongoing to improve prognosis [28]. It is noteworthy that we excluded from the analysis salivary glands tumors, already presented in a previous publication from our group [19], and head and neck sarcomas and mucosal melanomas.

CNAO synchrotron facility can currently offer both PT and CIRT, while new particle species, such as helium and oxygen will be available for clinical use soon. As previously reported, selection of beam quality at CNAO is customized [29] with CIRT mainly indicated for more radioresistant tumors to exploit the high radiobiological efficacy (such as salivary glands, sarcomas, and mucosal melanomas) [30] and PT for more radiosensitive epithelial tumors to spare toxicity, such as for tumors with SCC histology. The same philosophy also guides re-RT indication at CNAO, with CIRT as the first choice preferred for more aggressive and poorer prognosis hystotypes (such as salivary glands) and PT in case of CIRT judged not feasible because of a high risk of toxicity. Given the high stage and aggressive features at the recurrence presentation of the SNCs included in our series, the majority of the relapsed tumors were rechallenged with CIRT, even SCC, with the aim to improve outcomes since the preclinical evidence of intrinsic radioresistance of the relapsed tumor clones acquired after a first photon RT course [11]. Only for two patients with ONB and SNUC, re-RT was judged not feasible with CIRT, but PT was preferred since it was considered at high risk of post re-RT toxicity in case of CIRT, in consideration of the demolitive surgery with flap reconstruction before re-RT.

Patel et al. already reported advantages in the treatment of SNCs with particle therapy compared to photon RT in a metanalysis on different series at the primary treatment phase, although reporting higher neurological toxicity in the particle therapy series since the negative selective bias of worse prognosis patients referred for particle RT [31]. Concerning the issue of particle re-RT, not only well-designed prospective clinical trials are lacking but also randomized trials are currently missing to compare particle to photon RT and to assess the possible advantages of particle re-RT for SNCs. In a multicentric retrospective study on mixed head and neck tumor sites (including SNCs) from Japan, where CIRT for SNCs has been covered by the national insurance since January 2018 owing to the effectiveness [32], patients retreated with particle therapy (26 patients with PT or CIRT) had better outcomes compared to photon RT (150 with CyberKnife or IMRT) [33]. CIRT was indicated in a histology driven fashion for non-SCC tumors located in the skull base and head, while photons and PT were indicated for the other types of tumors.

Other authors have reported good outcomes of re-RT for SNCs using particle therapy. In this regard, Fan et al. in a subanalysis on SNCs retreated with PT described 2-year LC and OS rates of 77% and 66%, respectively, although in a publication including a mixed and inhomogeneous population, with most RT-naïve patients (79%) [17]. Furthermore, another mixed naïve/re-RT series of 42/27 SNCs form the USA treated with PT reported in the subanalysis for only re-RT cases, 3-year OS and freedom from disease progression (FFDP) and freedom from locoregional recurrence (FFLR) of 76.2%, 32.1%, and 33.8%, respectively [15]. Although a direct comparison is difficult since the inhomogeneity of the series, our study reported a poorer outcome in terms of 3-year OS compared to the listed publications but not for the PFS as compared to the latter study. Furthermore, different from our cohort where only re-RT patients were considered, Hu et al. included in their series 111 SNCs, but 81 were RT-naïve patients [16], with 2-year local progression-free survival (LPFS) and OS rates for the entire series of 83% and 82%, respectively. On the other side, Hayashi et al. analyzed a cohort of 48 head and neck relapsed patients, of whom 31 had SNCs of non-SCC origin and were reirradiated with CIRT after a first course of CIRT (different from our series where the first RT course was delivered with photon RT) with 2-year LC and OS rates of, respectively, 40.5 and 59.6% [20]. The peculiarities of the described studies might render difficult comparison with oncologic outcomes reported in our cohort but also might explain the differences.

In Held et al., it is reported the experience of the Heidelberg CIRT center HIT in Germany in a retrospective series of 229 patients with different histologies (26.2% SCC) relapsed at the head and neck in different locations, including at the paranasal sinuses, in 49/229 patients, and retreated at a median dose of 51 GyRBE with CIRT [34]. With a median follow-up time of 28.5 months, median LPFS after CIRT was 24.2 months, and median OS was 26.1 months. Our data confirms improvement in survival in case of re-RT with particle therapy, considering the median OS reported of our cohort of 35 months, although the small patient number probably negatively affected the LC in our series. The highlighted finding in Held et al. was the better LC at multivariate analysis for relapsed patients with ACC compared to the tumors of the other histological types [34], possibly further explaining the worst LC in our series, where ACC were intentionally not included in the analysis. For the future, well-designed prospective studies are warranted to assess the benefit of particle therapy for recurrent SNCs in the re-RT setting.

When considering our mild toxicity data with only G0–2 acute toxicity reported and no late toxicity higher than G3 (only 1 patient), we might conclude that re-RT for SNCs with particle therapy is safe and feasible. In this regard, a retrospective series from China from the SPHIC Shanghai Proton and Heavy Ion Treatment Center on a total of 141 patients, including SNCs with different histologies (SCC, salivary gland, and sarcomas), reported 10 cases of late severe effects with 4 deaths of hemorrhage secondary to soft tissue necrosis after re-RT with CIRT at 60 GyRBE median dose [35]. In comparison with the Chinese data, in our series only one case of soft tissue necrosis heading to oral fistula was reported in one SCC patient, which was managed with a conservative approach with antibiotic treatment in cycles to avoid extension of the necrotic front due to repeated infections. In this regard, our choice for CIRT has been to adopt the fractionation schedule of the German experience from HIT [34] (mainly 3 GyRBE/fr) and not the large dose/fr of the fractionation schedules derived from the Japanese experience to reduce the risks of ulceration and fistula formation because of the rapid and prompt tumor response after CIRT re-RT [11].

We are aware that our study has several limitations, in particular the small number of patients, the retrospective nature, the short follow-up, and the inhomogeneous mix of tumors with different characteristics, histologies, and treatment modalities. Only small cohorts with very heterogeneous histologies and locations in the head and skull base with short follow-up are available on particle-reirradiated patients, sometimes mixed with naïve patients. This is because SNCs are rare tumors, hadron therapy is a relative new type of treatment, and only few particle facilities treating patients are available in the world, especially with carbon ions (only 13).

## 5. Conclusions

Due to the small patients number in our study, it is difficult to draw any reliable conclusion on the benefit of particles in terms of efficacy and safety in the re-RT setting for SNCs. However, our preliminary data show acceptable toxicity and outcome results. Our report supports re-RT with particles and encourages treatment in a larger series of patients and studies designed in a prospective and multicenter manner with more stringent selection criteria. Considering the peculiarity of the re-RT setting, where the balance between efficacy and safety is delicate, it is important that re-RT is delivered in centers of high expertise, where it is possible to manage acute and long-term toxicities.

## Figures and Tables

**Table 1:** Patients and tumor characteristics at the time of primary treatment.

Patients Characteristics	All Patients (n = 15)
**Median age**	58 (range 32–84)
**Gender**	
Males	13 (86.7%)
Females	2 (13.3%)
**Histology**	
ITAC	7 (46.7%)
SCC	2 (13.3%)
ONB	3 (20%)
SNUC	2 (13.3%)
Adenocarcinoma	1 (6.7%)
**Stage (TNM VIII ed.)**	
I	0
II	2 (13.3%)
III	7 (46.7%)
IV	6 (40%)
**Site of primary tumor**	
Nasal cavity	7 (46.7%)
Ethmoid sinus	6 (40%)
Maxillary sinus	2 (13.3%)
**Primary treatment**	
Surgery	2 (13.3%)
Surgery + RT	8 (53.3%)
Exclusive RT	5 (33.4%)
**Type of first RT**	
Photons	14 (93.3%)
Gammaknife	1 (6.7%)
**First RT characteristics**	
Median dose	60 Gy (range 20–70)
Median dose/fraction (fr)	2 Gy/fr (1.8–20)

**Table 2:** Patients and tumor characteristics at the time of particle re-RT.

Patients Characteristics	All Patients (15)
**Median age**	61.5 years (range 38–87)
**Median time from first RT**	37 months (range 10–213)
**No. of relapses before the particle re-RT**	
1	5 (33.3%)
2	7 (46.7%)
3	3 (20%)
**Site of relapsed tumor at the particle re-RT**	
Maxillary sinus	2 (13.3%)
Ethmoid sinus	4 (26.6%)
Nasal cavity	7 (46.7%)
Sphenoid sinus	1 (6.7%)
Retromolar trigone	1 (6.7%)
**Pattern of failure after particle re-RT**	
Infield	9 (60%)
Marginal	6 (40%)
**Number of previous surgery**	
0	2 (13.3%)
1	7 (46.7%)
2	5 (33.3%)
3	1 (6.7%)
**Re-RT particle type**	
PT	2 (13.3%)
CIRT	13 (86.7%)
**Re-RT schedule**	
Median dose	54 GyRBE (range 45–64)
Median dose/fr	3 GyRBE
Median EQD2 (α/β = 3)	61.2 Gy
**Median GTV**	36.41 cm^3^ (3.4–122.89)
**Median follow-up time**	22 months (range 6–95)
**Acute Toxicity**	
G0	1 (6.7%)
G1-2	14 (93.3%)
G3-4	0
**Late Toxicity**	
G0	4 (26.6%)
G1-G2	10 (66.7%)
G3-G4	1 (6.7%)

**Table 3:** Acute and late toxicity details after particle re-RT.

	Number of Toxic Effects			
Grade of Toxic Effects	Grade 1	Grade 2	Grade 3	Grade 4
**ACUTE**				
Mucositis	2	4	0	0
Dermatitis	6	3	0	0
Edema	0	1	0	0
Conjunctivitis	2	2	0	0
Neuropathy	0	2	0	0
Dry mouth	0	1	0	0
**LATE**				
Dry mouth	1	1	0	0
Dysphagia	1	1	1	0
Neuropathy	0	3	0	0
Brain necrosis	1	2	0	0
Periorbital edema	0	1	0	0
Dry Eye	1	0	0	0
Soft tissue necrosis	0	1	0	0
Hypopituitarism	0	1	0	0
Alopecia	1	0	0	0
Fibrosis	1	0	0	0
Trismus	1	0	0	0

## Data Availability

The datasets used and/or analyzed during the current study are available from the corresponding author upon request and with compliance with the CNAO internal regulations for data sharing.
